# Rhizosphere bacterial and fungal community structure varies among field-grown *Mentha spicata* accession plots with different essential oil profiles

**DOI:** 10.3389/fmicb.2026.1939529

**Published:** 2026-09-03

**Authors:** Ákos Juhász, Yasmine Wazzani, Szilvia Tavaszi-Sárosi, Katalin Patonay, Ferenc Olasz, Katalin Posta

**Affiliations:** 1 Agribiotechnology and Precision Breeding for Food Security National Laboratory, Institute of Genetics and Biotechnology, Hungarian University of Agriculture and Life Sciences, Gödöllõ, Hungary; 2 Doctoral School of Natural Sciences, Hungarian University of Agriculture and Life Sciences, Gödöllõ, Hungary; 3 Department of Medicinal and Aromatic Plants, Institute of Horticultural Science, Hungarian University of Agriculture and Life Sciences, Budapest, Hungary; 4 Food and Wine Knowledge Centre, Eszterházy Károly Catholic University, Eger, Hungary

**Keywords:** 16S rRNA gene sequencing, aromatic plants, essential oil composition, ITS sequencing, *Mentha spicata*, rhizosphere microbiome

## Abstract

**Introduction:**

Aromatic plants produce diverse secondary metabolites, yet how within-species phytochemical variation relates to rhizosphere bacterial and fungal communities remains poorly resolved. *Mentha spicata* displays substantial essential oil (EO) compositional variation, providing a field system to examine rhizosphere microbiome structuring among accession plots.

**Methods:**

Five vegetatively propagated *M. spicata* accessions representing three operational EO-based groups (L-carvone group, Piperitenone oxide group, and Dihydrocarvone group) were examined under open-field conditions at full flowering. Each accession was grown in a single field plot, from which three spatially separated rhizosphere soil samples were collected; managed inter-row bulk soil was included as a contextual control. Soil physicochemical properties, aerial-tissue EO composition, and bacterial 16S ribosomal RNA gene and fungal ITS amplicon profiles were characterized. Bray-Curtis ordination, permutational multivariate analysis of variance, and distance-based redundancy analysis were used to assess community differences and associations with soil properties and accession-level EO profiles.

**Results:**

Bacterial communities were dominated by Actinomycetota, Pseudomonadota, Chloroflexota, Acidobacteriota and Bacillota, whereas fungal communities were dominated by Ascomycota, followed by Mortierellomycota and Basidiomycota. Fungal taxonomic profiles varied more strongly among groups, with J14 showing the highest Ascomycota proportion and Basidiomycota being most abundant in J7 and J11. Alpha-diversity differences were limited and, for bacteria, mainly reflected lower diversity in bulk soil. After excluding bulk soil, grouping by accession plot remained significant for bacteria (R^2^ = 0.6619, *p* = 0.0001) and fungi (R^2^ = 0.5060, *p* = 0.0001), with bacterial communities showing more compact structuring. Soil-based constrained models were significant for both microbial groups. pH and nitrate + nitrite nitrogen were significantly associated with bacterial and fungal community composition, while humus content was additionally significant for fungi. In contrast, accession-level models based on aerial-tissue EO gradients were not significant for bacteria (*p* = 0.342) or fungi (*p* = 0.317).

**Discussion:**

Field-grown *M. spicata* accession plots with contrasting aerial-tissue EO profiles harbored differentiated rhizosphere bacterial and fungal communities, and community variation was significantly associated with local soil properties. A statistically supported relationship with aerial-tissue EO composition was not detected, and these profiles are best regarded as accession-level chemical descriptors rather than direct rhizosphere drivers.

## 1 Introduction

The genus *Mentha* includes economically important aromatic and medicinal plants cultivated for their essential oils (EOs), phenolic compounds, culinary value, and biological activities. Among them, *Mentha spicata* L. (spearmint) is one of the most widely used taxa, valued in the food, cosmetic, and pharmaceutical sectors and frequently investigated for antioxidant and antimicrobial properties (Salehi et al., 2018; Sfaxi et al., 2025; Tavaszi-Sárosi et al., 2025). The biological and commercial value of spearmint is closely linked to its EO composition, which varies substantially among accessions, cultivars, environmental conditions, and phenological stages (Kokkini and Vokou, 1989; Telci et al., 2010; Kowalczyk et al., 2021; Zekri et al., 2023). This chemical variability is especially relevant because *M. spicata* does not represent a single, uniform EO profile. Although L-carvone- and limonene-rich oils are often associated with the typical spearmint aroma, several additional chemical profiles have been described, including linalool-rich, piperitenone oxide/piperitone oxide-rich, pulegone/piperitone-rich, dihydrocarvone-related, and other structurally distinct monoterpene-dominated types (Kokkini and Vokou, 1989; Kokkini et al., 1997; Baser et al., 1999; Tavaszi-Sárosi et al., 2025). Such chemotype variation reflects differences in monoterpene biosynthesis and provides a useful phenotypic framework for comparing plant accessions. However, EO profiles measured from aerial tissues should not be interpreted as direct measurements of rhizosphere metabolites; rather, they can be used as accession-level chemical descriptors that may reflect broader metabolic differences among plants.

The rhizosphere is a highly active soil compartment shaped by the continuous exchange of compounds between plant roots, soil, and microorganisms (Bulgarelli et al., 2013; Philippot et al., 2013). Root-derived carbon inputs, including sugars, amino acids, organic acids, phenolics, terpenoid-related compounds, and other secondary metabolites, can influence the colonization and reproduction of microorganisms and the formation of microbial communities (Pang et al., 2021; Koprivova and Kopriva, 2022). In turn, rhizosphere bacteria and fungi contribute to nutrient cycling, stress tolerance, pathogen suppression, and plant performance (Berendsen et al., 2012; Philippot et al., 2013). The resulting community structure is therefore shaped by both plant-associated factors, such as genotype, developmental stage and root exudation, and by environmental factors, including soil pH, organic matter, moisture, and nutrient availability (Bulgarelli et al., 2013; Genitsaris et al., 2020; Deng et al., 2024; Li L. et al., 2025).

Medicinal and aromatic plants are particularly interesting in this context because they produce chemically complex secondary metabolites with antimicrobial, allelopathic, or biostimulatory potential. Plant-derived compounds and residues may enter the soil through root exudation, decaying leaves, stem residues, rain wash-off, or volatile emissions, potentially modifying microbial biomass and community composition (Ainalidou et al., 2021; Zharkova et al., 2023). Soils can act as both sources and sinks of biogenic volatile organic compounds (VOCs), with decomposing litter, roots, and microorganisms contributing to belowground VOC pools and interactions (Peñuelas et al., 2014). For example, decaying tissues of *M. spicata* and *M. × piperita* were shown to stimulate soil microbial biomass and favor certain microbial groups in a plant-growth bioassay, whereas other aromatic species produced more suppressive effects (Ainalidou et al., 2021). In a related soil-amendment experiment, EO content in spearmint and peppermint residues decreased rapidly during decomposition, and EO composition shifted markedly over time, indicating that aromatic plant residues may provide dynamic rather than static chemical inputs to soil (Karamanoli et al., 2018). In monoculture systems, medicinal and aromatic crops, including *M. arvensis*, have also been associated with shifts in soil microbial richness, evenness, and community state, with partial restoration possible through intercropping or organic amendments (Misra et al., 2019). These findings suggest that aromatic plants can influence soil microbial communities, but the direction and magnitude of these effects depend strongly on plant identity, chemical profile, soil conditions, and management history.

Several studies on Lamiaceae and other medicinal plants support the idea that host identity and plant chemical traits may be associated with distinct rhizosphere or plant-associated microbiota. In Lamiaceae field systems, bacterial communities differed among oregano, peppermint, thyme, creeping thyme, and sage, with plant-specific rhizosphere effects and differential enrichment of major bacterial phyla (Zharkova et al., 2023). In *Thymus* spp., cultivable bacteria from leaves, roots, and rhizosphere showed compartment-specific patterns and differential tolerance to essential oils, suggesting that plant volatile chemistry may act as a selective factor, although the strongest evidence was observed for leaf-associated bacteria (Checcucci et al., 2017). Similar links between EO composition and cultivable bacterial assemblages have been explored in *Origanum* species, where different plant compartments and EO profiles were evaluated together (Castronovo et al., 2020; Semenzato et al., 2023). In other medicinal or Lamiaceae species, such as *Coleus barbatus*, *Salvia miltiorrhiza*, *Scutellaria baicalensis*, *Lavandula angustifolia*, and Mediterranean medicinal plant species, rhizosphere microbial communities were associated with developmental stage, soil properties, or plant metabolite profiles (Applebaum et al., 2022; Jamwal et al., 2023; Deng et al., 2024; Steinberger et al., 2024; Li L. et al., 2025; Li Y. et al., 2025).

Despite these advances, comparatively little is known about within-species rhizosphere microbial variation among *M. spicata* accessions with contrasting EO profiles. Most previous studies on *Mentha* have focused on EO composition, antimicrobial activity, agronomic performance, or mycorrhizal effects on plant growth and EO yield, rather than on bacterial and fungal community structure in the rhizosphere (Karagiannidis et al., 2011; Heydarizadeh et al., 2013; Adamović et al., 2025; Kowalczyk et al., 2021; Sfaxi et al., 2025). Moreover, studies that connect aromatic plant chemical variation with microbiome composition remain rare, and available examples often address different plant species, plant compartments, or cultivable bacterial fractions rather than high-throughput profiling of both bacterial and fungal rhizosphere communities (Checcucci et al., 2017; Castronovo et al., 2020; Semenzato et al., 2023).

A field comparison of *M. spicata* accessions grown under similar open-field conditions offers an opportunity to examine whether within-species phytochemical differentiation is accompanied by rhizosphere microbiome structuring. Such a design cannot eliminate microscale soil heterogeneity, but it reduces major environmental variation compared with multi-site surveys and provides a shared agronomic background for comparing the five accession plots. At the same time, soil physicochemical properties must be considered because even local differences in pH, humus content, or nutrient availability may contribute to bacterial and fungal community structure.

In this study, we investigated the rhizosphere bacterial and fungal communities of five field-grown *M. spicata* accessions representing three operational EO-based groups: L-carvone group, Piperitenone oxide group, and Dihydrocarvone group. Our objectives were to: (i) characterize the soil physicochemical background and aerial-tissue EO profiles of the accessions; (ii) compare bacterial and fungal diversity, taxonomic composition, and community structure among the five accession plots, with managed inter-row bulk soil included as a contextual control; and (iii) evaluate associations between microbial community composition, soil properties, and accession-level EO profiles. We hypothesized that field-grown *M. spicata* accession plots with contrasting EO profiles would harbor partially distinct rhizosphere bacterial and fungal assemblages, and that these patterns would be associated with local soil physicochemical variation and potentially with accession-level EO profiles.

## 2 Materials and methods

### 2.1 Experimental design and sampling

Plant and rhizosphere-enriched, root-associated soil samples were collected in late July 2025 from the experimental field of the Department of Medicinal and Aromatic Plants, Hungarian University of Agriculture and Life Sciences, at the full flowering stage. The stands had been established in 2022 by vegetative propagation from mother plants. Five *Mentha spicata* accessions representing three operational EO-based groups were included in the study: B1 (L-carvone group), B4 and J14 (Piperitenone oxide group), and J7 and J11 (Dihydrocarvone group). Capitalized names followed by “group” denote these operational EO-based categories, whereas lowercase compound names refer to individual EO constituents. Where the term chemotype is retained, it refers to this operational grouping based on the dominant EO constituents detected in aerial plant material, rather than to genetically confirmed or fixed chemotypic differentiation. The accession codes correspond to entries in the institutional gene bank. Each accession was grown in a single 1.5 × 2.5 m plot, with adjacent plots separated by 1.5 m-wide inter-row strips. These inter-row areas were regularly maintained by mechanical weeding or shallow tillage and therefore remained free of both cultivated plants and spontaneous vegetation. The field layout was not randomized or blocked, and no independent plot-level replication was available. The term “accession” denotes the plant material identified by its gene-bank code, whereas “accession plot” denotes the single field plot in which that accession was grown.

For each accession, three spatially separated sampling points were positioned approximately 1 m apart in an approximately triangular arrangement. At each sampling point, a block of vegetation and root-bearing soil measuring approximately 15 × 15 cm was excavated to a depth of 10–15 cm. The mint stands were dense and interconnected by extensive stolon networks; therefore, individual plants could not be reliably distinguished, and each excavated block represented an integrated local root system rather than a single plant. The soil dislodged by shaking the excavated root system was collected in its entirety. Loosely and tightly root-adhering soil fractions were not separated because the sandy loam soil adhered only weakly to the roots. Accordingly, the collected material is referred to as a rhizosphere-enriched, root-associated soil sample; the three samples from each accession plot were kept separate and were not pooled. For brevity, these samples are hereafter referred to as rhizosphere soil samples. They represent within-plot spatial variation and are not independent plot-level biological replicates.

Three separate bulk soil samples were collected as contextual controls from the vegetation-free inter-row strips located approximately 50 cm from the nearest plant rows. Because these strips were regularly disturbed by shallow cultivation during the growing season, the bulk samples represented managed inter-row soil rather than undisturbed natural soil. These samples are hereafter referred to as bulk soil. To minimize cross-contamination, sampling tools were decontaminated with 70% ethanol between sampling points. A fresh pair of disposable gloves was worn for each sampling point and additionally disinfected with 70% ethanol before sample handling. All soil samples were transferred directly into sterile 50 mL Falcon tubes, transported to the laboratory on ice, and processed upon arrival. Each soil sample was thoroughly mixed, and visible roots and other plant debris were removed before subsampling. For DNA-based analyses, 0.1 g aliquots of the homogenized soil were transferred into sterile 1.5 mL tubes and stored at −80 °C until extraction.

In parallel with soil sampling, aerial plant material for EO analysis was collected and pooled at the accession-plot level. Plant material was collected independently of the three soil sampling points and therefore was not paired with individual rhizosphere soil samples. The resulting EO profile therefore represents an accession-plot-level aerial-tissue descriptor rather than a direct measurement of root exudates, rhizosphere metabolites, or the chemical environment of the rhizosphere.

### 2.2 Soil physicochemical properties

The three rhizosphere soil samples from each accession plot and the three bulk soil samples were analyzed separately, with one measurement per parameter for each sample. Analyses were performed using standard Hungarian soil analytical methods. Soil physical classification was based on the Arany-type plasticity index. Soil pH was measured in 1 M KCl suspension, and humus content was determined by potassium dichromate oxidation. Available phosphorus (P_2_O_5_), sodium (Na^+^), and potassium (K_2_O) were extracted using ammonium lactate solution and determined as agronomically available or exchangeable nutrient fractions. Nitrate and nitrite nitrogen (NO_3_^–^ + NO_2_^–^) were extracted with 1 M KCl and quantified according to standard soil analytical procedures.

### 2.3 Essential oil extraction and GC-MS analysis

For essential oil extraction, approximately 200 g of flowering shoots (about 40 cm in length) were harvested from each accession and pooled at the accession-plot level before being shade-dried for 3 weeks. After drying, the leaves were separated from the stems and subjected to hydrodistillation using a Clevenger-type apparatus. For each accession, three hydrodistillations were performed, each using a separate 10 g aliquot of the pooled dried leaf material and 500 mL of distilled water for 2.5 h. Because all aliquots originated from the same accession-level pooled plant material, the three hydrodistillations did not constitute independent plant or plot replicates. Residual water was removed from the obtained essential oils using anhydrous sodium sulfate, and the oils were stored in airtight vials at 4 °C until analysis.

The essential oil obtained from each of the three hydrodistillations was analyzed separately by gas chromatography-mass spectrometry (GC-MS) using an Agilent Technologies 6890N gas chromatograph coupled to a 5975 inert mass selective detector and equipped with an HP-5MS capillary column (30 m × 0.25 mm i.d., 0.25 μm film thickness). The oven temperature was initially set to 60 °C, then increased at 3 °C/min to 240 °C and held at the final temperature for 5 min. Helium was used as the carrier gas at a constant flow rate of 1 mL/min. The injector and detector temperatures were both set to 250 °C, and the split injection ratio was 30:1. EO samples were diluted in n-hexane (10 μL EO in 1 mL solvent), and 0.2 μL of the diluted sample was injected.

Compound identification was based on linear retention indices (LRI) calculated relative to a homologous series of n-alkanes (C9-C23) using the Van den Dool and Kratz equation (Van Den Dool and Kratz, 1963), comparison with literature retention index data, and mass spectral matching against commercial libraries (NIST and Wiley). The identified constituents were consistent with previously reported *Mentha* EO components.

### 2.4 DNA extraction and amplicon sequencing of soil samples

Genomic DNA was extracted separately from 100 mg aliquots of each rhizosphere and bulk soil sample using the ZymoBIOMICS 96 MagBead DNA Kit (Zymo Research, Irvine, CA, USA), following the manufacturer’s instructions. DNA concentration was measured using the Quant-iT dsDNA BR Assay Kit (Thermo Fisher Scientific, Waltham, MA, USA) on an Infinite Pro 200 F Nano+ fluorescent plate reader (Tecan, Männedorf, Switzerland).

Bacterial and fungal community composition was assessed by high-throughput amplicon sequencing of the bacterial 16S rRNA gene and the fungal internal transcribed spacer (ITS) region, respectively. Amplicon library preparation and sequencing were performed by Xenovea Kft. (Szeged, Hungary) on an Illumina NextSeq 2000 platform using 2 × 300 bp paired-end chemistry. The V3-V4 region of the bacterial 16S rRNA gene was amplified using tagged primers described previously (Klindworth et al., 2013). Fungal ITS regions were amplified using tagged ITS1F and ITS4R primers following standard fungal metabarcoding library preparation protocols.

Briefly, 50 ng of template DNA was used in the initial amplification step (PCR1), which was performed with KAPA HiFi HotStart Ready Mix (Roche AG, Basel, Switzerland) for 25 cycles. PCR products were purified, and 10 ng of the purified amplicons was used as input for indexing PCRs with the Nextera XT Index v2 Kit (Illumina, San Diego, CA, USA) and KAPA HiFi HotStart Ready Mix for 8 amplification cycles. Indexed libraries were purified using 0.8 × volume KAPA Pure Beads (Roche AG, Basel, Switzerland). Library quantification was performed with the Equalbit 1 × dsDNA HS Assay Kit (Vazyme Biotech, China) using the same fluorescent plate reader. Library size distribution was evaluated by capillary electrophoresis using a LabChip GX Touch HT Nucleic Acid Analyzer and the DNA NGS 3K Assay Kit (Revvity, Waltham, MA, USA). Libraries were normalized to equimolar concentrations, pooled, and sequenced on the Illumina NextSeq 2000 platform.

### 2.5 Bioinformatic processing

Raw sequencing reads with a minimum depth of 100,000 reads per sample were demultiplexed and adapter-trimmed using the NextSeq Control Software. Reads with a mean quality score below 30 were discarded using the FastQ Toolkit (Illumina, Inc., San Diego, CA, USA). Paired-end reads were then processed in the FROGS pipeline v4.2.0 (Escudié et al., 2018). Forward and reverse reads were quality-filtered and merged with VSEARCH v2.17.0 (Rognes et al., 2016), retaining amplicons between 150 and 600 bp and allowing a mismatch rate of 0.1. Merged sequences were clustered using SWARM v3.0.0 (Mahé et al., 2014) with an aggregation distance of one nucleotide (d = 1) and fastidious refinement. Chimeric sequences were removed separately within each sample using the remove_chimera.py script implemented in FROGS. The term ASV (amplicon sequence variant) refers to these SWARM-derived sequence clusters, following the terminology used in FROGS outputs, rather than to exact sequence variants inferred by a denoising algorithm. Clusters representing fewer than 0.005% of the total read count were excluded from downstream analyses. No additional abundance- or prevalence-based filtering was applied. Fungal sequences were screened with ITSx using the Fungi profile while retaining conserved regions. For bacterial data, only clusters assigned to Bacteria or Archaea were retained; chloroplast, mitochondrial, eukaryotic, and other non-target clusters were removed. Clusters unresolved at lower taxonomic ranks were retained and labeled according to the lowest resolved rank. Taxonomic assignment was performed using BLAST v.2.16.0 (McGinnis and Madden, 2004) against the SILVA 138.2 database (Quast et al., 2012) for bacterial 16S rRNA sequences and the UNITE Fungi 10.0 database (Abarenkov et al., 2024) for fungal ITS sequences. No separate species-level confidence threshold was applied. Prior to alpha- and beta-diversity analyses, ASV abundance tables were rarefied to the sequencing depth of the sample with the lowest read count. Accordingly, bacterial 16S rRNA datasets were rarefied to 107,795 reads per sample, whereas fungal ITS datasets were rarefied to 58,383 reads per sample. The same filtered and rarefied abundance tables were used for both alpha- and beta-diversity analyses. No samples were excluded because of insufficient sequencing depth.

### 2.6 Statistical analysis

Statistical analyses were performed in R version 4.4.3 (Fox and Leanage, 2016), and *p* < 0.05 was considered statistically significant. Differences in soil physicochemical properties among the five accession plots and bulk soil were assessed using one-way analysis of variance (ANOVA), followed by Tukey’s HSD *post hoc* test. Alpha diversity indices, including Observed ASVs, Chao1 richness, Shannon diversity, and inverse Simpson diversity, were calculated using the Phyloseq v1.38.0 package within the FROGS framework (McMurdie and Holmes, 2013). Differences in alpha diversity among the sampled groups were assessed using one-way ANOVA followed by Tukey’s HSD *post hoc* test.

Microbial community structure was visualized using principal coordinate analysis (PCoA) based on Bray-Curtis dissimilarities. PERMANOVA was conducted with 9999 permutations to test differences in community composition among all sampled groups. The analysis was subsequently repeated after excluding bulk soil to evaluate differences among the five accession plots. Homogeneity of multivariate dispersion was assessed for the corresponding groupings using PERMDISP with the betadisper function in the vegan package, followed by permutation testing with 9999 permutations. Because the three rhizosphere samples from each accession plot represented within-plot spatial variation rather than independent plot-level replicates, comparisons among accessions were interpreted as differences among the sampled accession plots rather than as independently replicated accession effects.

Taxonomic composition was evaluated using relative abundances calculated from the filtered, non-rarefied ASV tables. Family-level heatmaps were generated using the ComplexHeatmap package after row-wise z-score transformation.

Bray-Curtis-based distance-based redundancy analysis (dbRDA) was used to evaluate associations between bacterial and fungal community composition and either measured soil physicochemical variables or accession-level aerial-tissue EO profiles in rhizosphere samples from the five M. spicata accession plots. Bulk soil samples were excluded from these analyses. Prior to soil dbRDA, multicollinearity among the soil physicochemical variables was evaluated using variance inflation factors (VIFs). Available potassium was excluded from the constrained models because its VIF exceeded 10 (VIF = 10.36), whereas the VIFs of all remaining variables were below 4. Potassium was retained in the descriptive analyses but was not included as an explanatory variable in the dbRDA models. The final soil models therefore included humus content, pH, nitrate + nitrite nitrogen, available sodium, and available phosphorus. Soil variables were standardized before analysis. Bray-Curtis-based dbRDA was performed separately for bacterial and fungal communities using Lingoes correction. The significance of the overall models, individual constrained axes, and marginal contribution of each explanatory variable was evaluated using 9999 permutations. Model R^2^ and adjusted R^2^ values were calculated for the final models.

The EO analysis was restricted to the ten compounds that reached more than 5% relative abundance in at least one EO sample. For each accession, the mean relative abundance of these compounds was calculated from the three analytical determinations. Because the resulting ten-component predictor set was strongly collinear and unsuitable for an accession-level constrained model, the accession-level EO profiles were Hellinger-transformed and subjected to principal component analysis (PCA). The first two principal components were retained because together they represented 94.0% of the variation in EO composition. Bacterial and fungal relative-abundance profiles were averaged across the three rhizosphere samples of each accession, resulting in five accession-level microbial community profiles. Separate Bray-Curtis-based dbRDA models were then fitted for bacterial and fungal communities using EO-PC1 and EO-PC2 as explanatory variables and Lingoes correction. Model significance, constrained axes, and marginal effects of the two EO-PCs were evaluated using all 119 possible non-identity permutations of the five accession-level observations. Model R^2^, adjusted R^2^, and VIF values were calculated. EO-PC1 and EO-PC2 were displayed as biplot vectors in the dbRDA ordinations.

## 3 Results

### 3.1 Soil physicochemical properties

All soil samples were classified as sandy loam, indicating broadly similar texture across the rhizosphere and bulk soils. Despite this overall similarity, several chemical parameters differed significantly among the sampled groups (Table 1). Humus content ranged from 1.53% in J11 to 2.18% in B1, with J7 also showing relatively high values, whereas B4, J14, and the bulk soil were intermediate. Soil pH varied only within a narrow alkaline range (7.94–8.02), although small but significant differences were still detected. Nitrate + nitrite nitrogen content showed marked variation and was highest in J14, while the other rhizosphere and bulk soil samples had substantially lower values. Available sodium also varied among groups, with the highest mean values recorded in the bulk soil and B1, and the lowest in J11. Available phosphorus and potassium varied significantly as well: B1 had the highest concentrations of both nutrients, whereas J11 showed the lowest potassium level and, together with J7, the lowest phosphorus values.

**TABLE 1:** Physicochemical properties of rhizosphere soil from five *M. spicata* accession plots and bulk soil.

Parameter	B1	B4	J7	J11	J14	Bulk
Humus content (%)	2.18 ± 0.16a	1.88 ± 0.04b	2.02 ± 0.10ab	1.53 ± 0.04c	1.91 ± 0.10b	1.86 ± 0.07b
pH (KCl)	7.96 ± 0.03bc	7.99 ± 0.02ab	7.94 ± 0.02c	7.94 ± 0.02bc	7.95 ± 0.02bc	8.02 ± 0.02a
(NO_3_^–^ + NO_2_^–^) (mg/kg)	2.52 ± 0.09b	1.86 ± 0.27c	1.99 ± 0.10bc	2.08 ± 0.07bc	7.25 ± 0.38a	1.91 ± 0.05c
Available Na^+^ (mg/kg)	51.27 ± 4.95a	44.37 ± 6.48ab	46.17 ± 4.82ab	36.53 ± 2.81b	47.60 ± 3.44ab	52.03 ± 3.76a
Available P (P_2_O_5_ mg/kg)	740.67 ± 29.57a	647.67 ± 73.90ab	600.67 ± 35.13b	593.00 ± 19.29b	633.67 ± 70.85ab	693.00 ± 26.89ab
Available K (K_2_O mg/kg)	226.00 ± 25.51a	142.67 ± 20.60b	115.33 ± 12.74bc	26.23 ± 0.38d	83.27 ± 6.36c	111.33 ± 6.66bc

Values represent means ± standard deviation based on three soil samples for each accession plot and for bulk soil (*n* = 3). Different lowercase letters within a row indicate significant differences among groups according to one-way ANOVA followed by Tukey’s HSD test (*p* < 0.05). EO-based groups: B1, L-carvone group; B4 and J14, Piperitenone oxide group; J7 and J11, Dihydrocarvone group.

### 3.2 Essential oil composition

**FIGURE 1:**
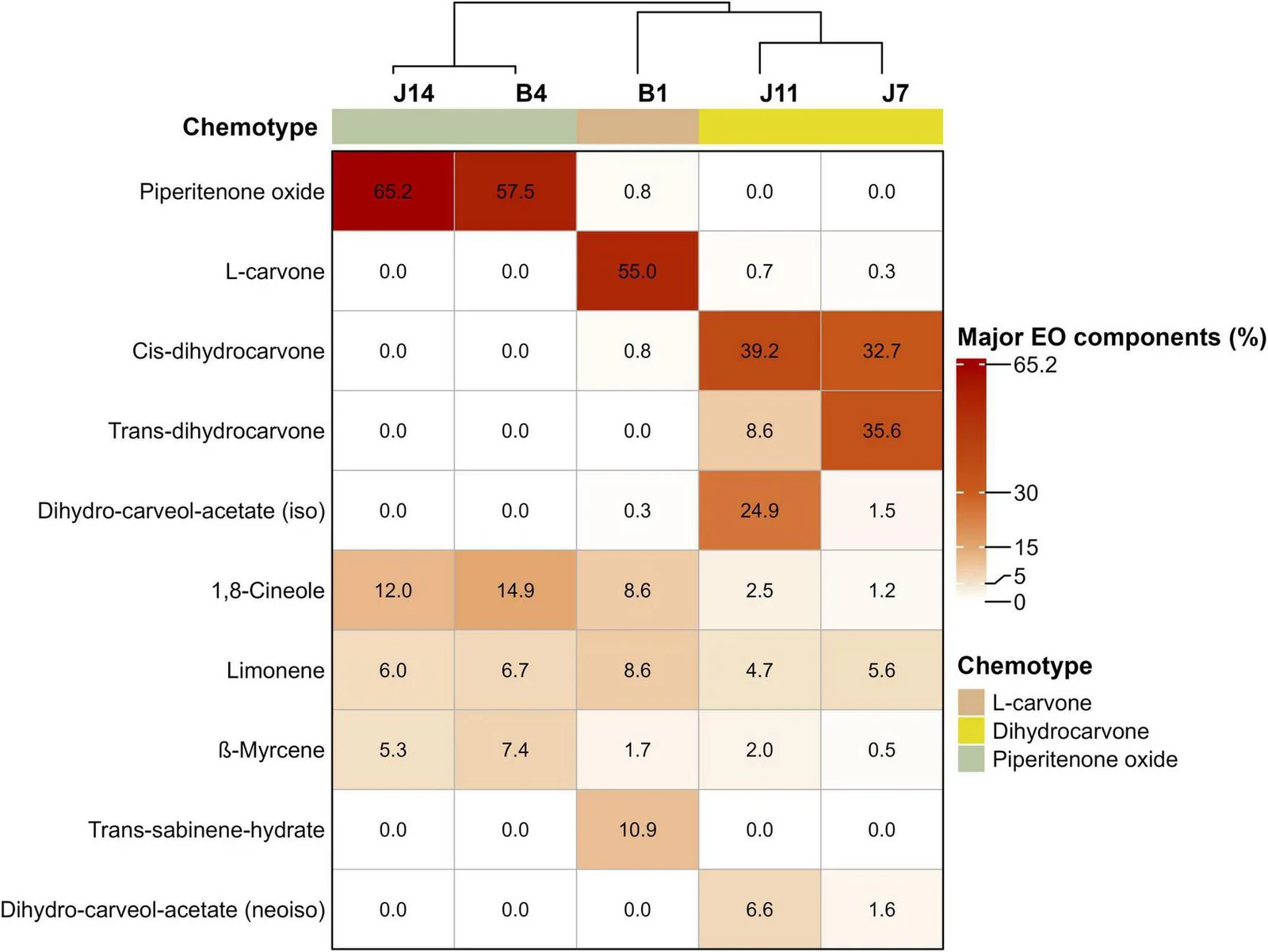
Heatmap of major EO components in the five *M. spicata* accessions. Only compounds with a mean relative proportion exceeding 5.0% in at least one accession are shown. Cell values represent mean relative percentages obtained three hydrodistillations performed on separate aliquots of the same pooled leaf material from each accession plot.

EO composition results of the analyzed spearmint accessions are presented in Figure 1 and Supplementary Table 1. *M. spicata* J14 and B4 were assigned to the Piperitenone oxide group, with piperitenone oxide accounting for 65.2 and 57.5% of the EO composition, respectively. The second most abundant component in these accessions was 1,8-cineole, at 12.0% in J14 and 14.9% in B4. B1 represented the L-carvone group, with L-carvone accounting for 55.0% of the EO composition. *M. spicata* J11 and J7 comprised the Dihydrocarvone group. Cis-dihydrocarvone was a major component in both accessions, accounting for 39.2% in J11 and 32.7% in J7. Trans-dihydrocarvone accounted for 8.6% in J11 and 35.6% in J7, whereas dihydro-carveol-acetate (iso) reached 24.9% in J11 but only 1.5% in J7.

### 3.3 Sequencing output and ASV richness

Amplicon sequencing was performed on rhizosphere soil from the five *M. spicata* accession plots and on bulk soil, with three soil samples for each accession plot and for bulk soil. After quality filtering, the bacterial 16S dataset comprised 2,547,611 sequences grouped into 3157 ASVs, while the fungal ITS dataset comprised 1,796,824 sequences grouped into 837 ASVs. Across the full bacterial dataset, 28 phyla, 65 classes, 136 orders, 204 families, 435 genera, and 527 species were detected (Supplementary Table 2). The corresponding fungal dataset contained 12 phyla, 43 classes, 81 orders, 156 families, 272 genera, and 379 species (Supplementary Table 3). Bacterial ASV richness was relatively consistent across rhizosphere samples, ranging from 2973 to 3122, whereas bulk soil samples showed lower values of 2648–2872. Fungal ASV counts were more variable, ranging from 240 to 478 across the dataset.

The ASV counts and taxonomic summaries reported in this section are based on the full quality-filtered dataset. Taxonomic composition analyses presented below were based on relative abundances calculated from the filtered, non-rarefied ASV tables, whereas alpha- and beta-diversity analyses were performed using the rarefied ASV tables.

### 3.4 Taxonomic composition of bacterial and fungal communities

**FIGURE 2:**
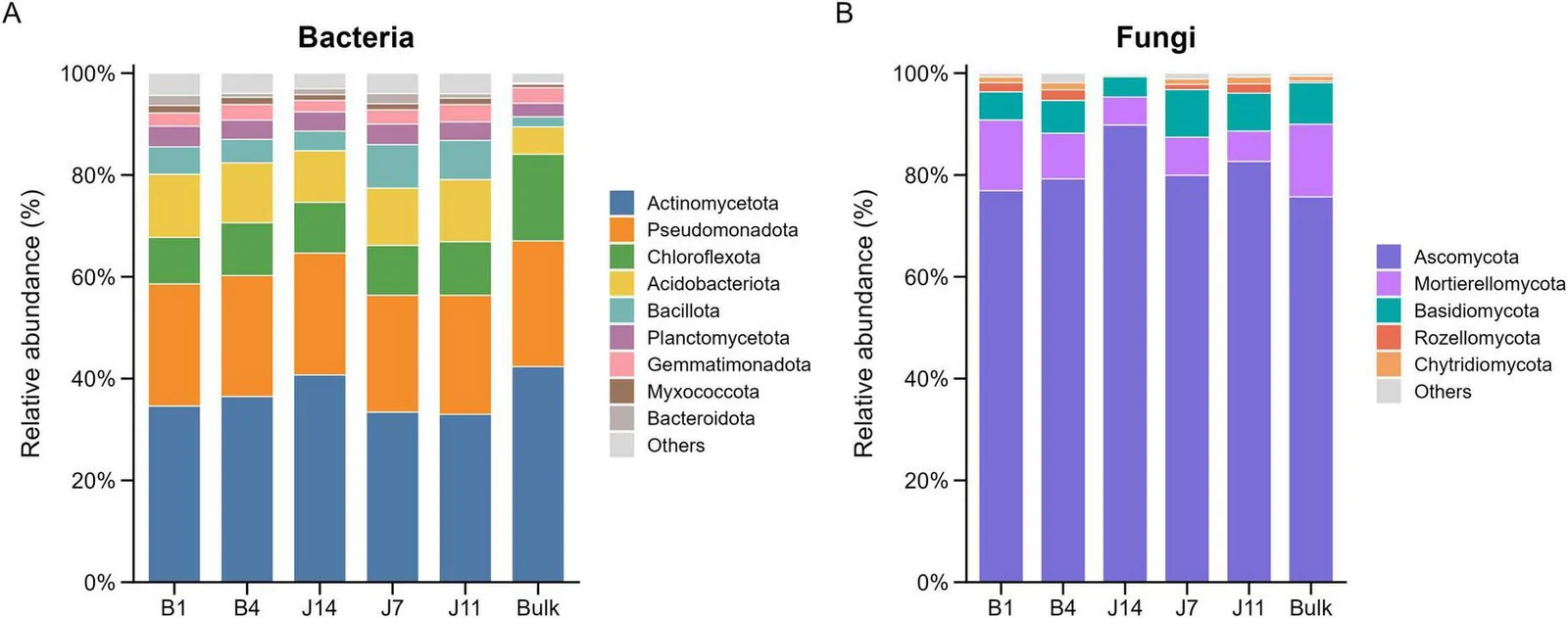
Relative abundance of bacterial **(A)** and fungal **(B)** communities at phylum level across rhizosphere soil from the five *M. spicata* accession plots and bulk soil. Only phyla with a mean relative abundance exceeding 1% across all samples are shown separately; the remaining phyla are grouped as “Others.” Values are based on non-rarefied relative abundance data averaged across three soil samples for each accession plot and for bulk soil.

At the phylum level, bacterial communities were dominated by Actinomycetota, Pseudomonadota, Chloroflexota, Acidobacteriota, and Bacillota, with mean relative abundances of 36.76%, 23.79%, 11.14%, 10.53%, and 5.34%, respectively, across all samples (Figure 2A). Together, these five phyla accounted for 87.56% of the total bacterial community. The overall bacterial phylum composition was relatively similar among the five accession plots, although some consistent differences were observed. Bulk soil showed the highest relative abundance of Actinomycetota (42.35%) and Chloroflexota (17.03%), but the lowest relative abundance of Acidobacteriota (5.36%) and Bacillota (1.99%). In contrast, Acidobacteriota was more abundant in all rhizosphere samples, ranging from 10.14% in J14 to 12.41% in B1. Bacillota was most abundant in J7 and J11 rhizosphere samples, both belonging to the Dihydrocarvone group, reaching 8.55 and 7.67%, respectively.

Fungal communities were dominated by Ascomycota, which represented 80.71% of the total fungal community across all samples (Figure 2B). The next most abundant fungal phyla were Mortierellomycota (9.35%), Basidiomycota (6.81%), Rozellomycota (1.23%), and Chytridiomycota (1.02%), while all remaining phyla together accounted for less than 1% of the fungal community. Fungal phylum-level profiles varied more among the sampled groups than bacterial profiles. J14 had the highest relative abundance of Ascomycota (89.82%), whereas B1 and bulk soil showed lower Ascomycota proportions and relatively higher Mortierellomycota abundances (13.88 and 14.27%, respectively). Basidiomycota was most abundant in rhizosphere samples from J7 and J11 in the Dihydrocarvone group, where it reached 9.34 and 7.45%, respectively.

**FIGURE 3:**
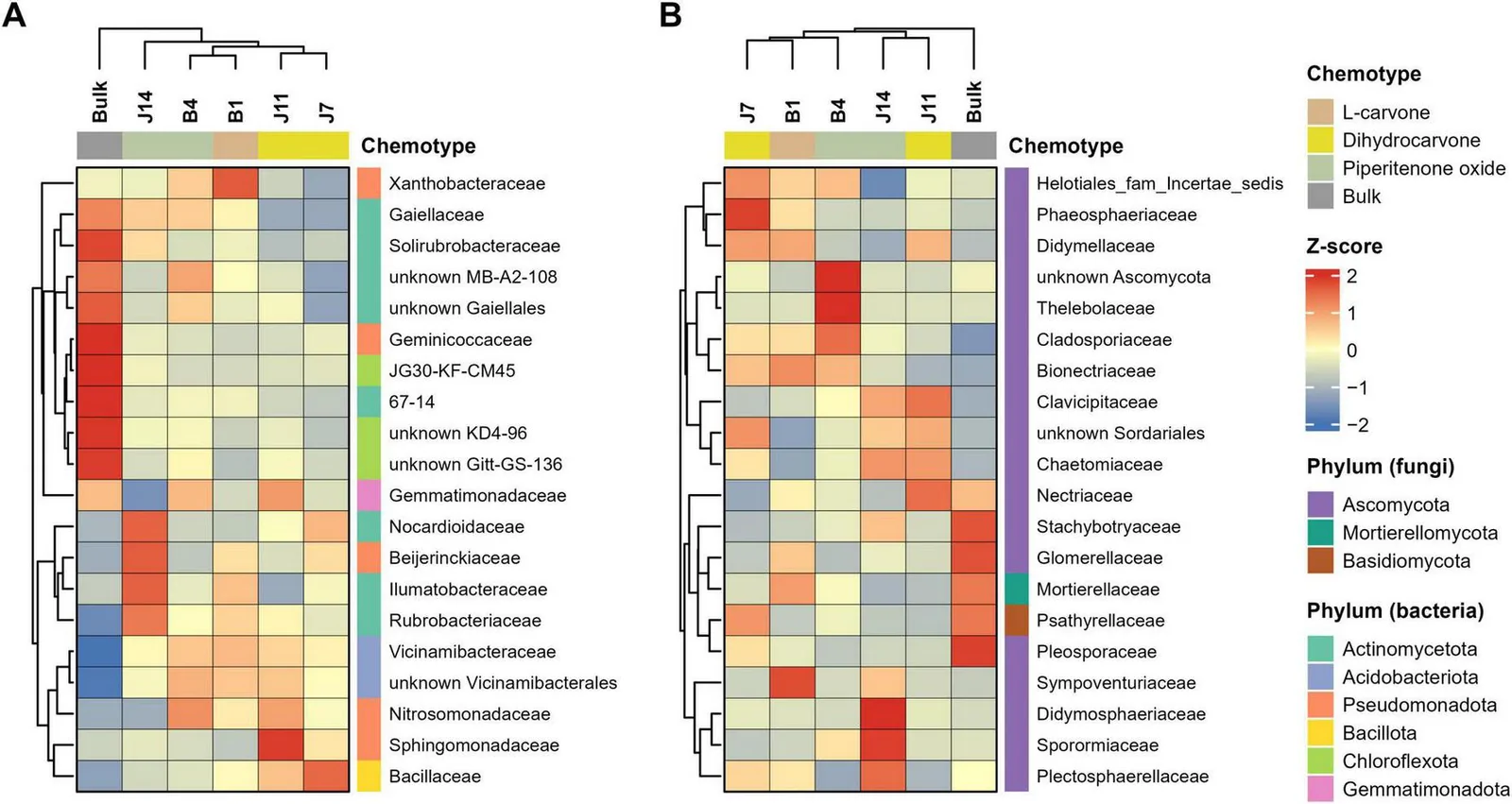
Heatmap of the 20 most abundant bacterial **(A)** and fungal **(B)** families. The heatmap color scale represents row-wise z-score-transformed values calculated from non-rarefied relative abundances. Values are based on mean relative abundances across three soil samples for each accession plot and for bulk soil.

Family-level composition was further examined using the 20 most abundant bacterial and fungal families (Figure 3). In the bacterial dataset, no single family dominated the community. The most abundant family-level groups across all samples were unknown Vicinamibacterales (4.12%), unknown Gaiellales (3.80%), unknown KD4-96 (3.73%), Geminicoccaceae (3.53%), Nocardioidaceae (3.49%), *67-14* (3.46%), and Bacillaceae (3.37%). Bulk soil differed from the rhizosphere samples by showing higher relative abundances of several family-level groups, particularly Geminicoccaceae, unknown *KD4-96*, and *67-14*. Among the rhizosphere samples, J7 showed the highest Bacillaceae abundance (6.09%), consistent with the elevated Bacillota proportion observed at the phylum level.

Fungal family-level composition was more uneven and more variable among the sampled groups. The dominant fungal families across the dataset were Nectriaceae (15.02%), Chaetomiaceae (12.44%), Mortierellaceae (9.16%), Stachybotryaceae (8.22%), and Didymellaceae (8.14%). Nectriaceae reached its highest abundance in J11 (20.43%), Stachybotryaceae was most abundant in bulk soil (15.91%), and Didymellaceae was particularly abundant in J7 (13.37%).

### 3.5 Alpha diversity of bacterial and fungal communities

**FIGURE 4:**
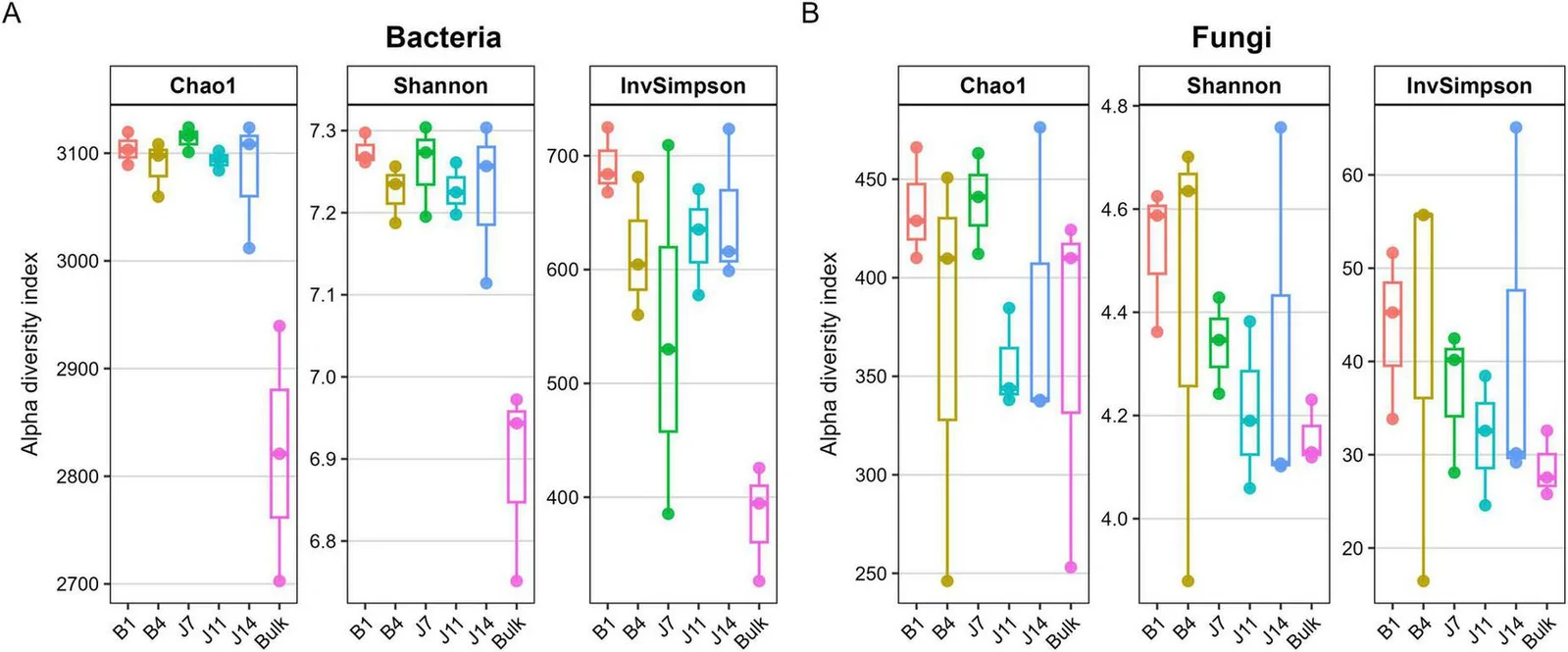
Alpha diversity of bacterial **(A)** and fungal **(B)** communities. Boxplots show Chao1 richness, Shannon diversity, and inverse Simpson diversity calculated from rarefied ASV abundance data. Points represent individual soil samples (*n* = 3 for each accession plot and for bulk soil). Statistical results for Observed ASVs and the plotted indices are provided in Supplementary Tables 4, 5.

Bacterial alpha diversity differed significantly among the sampled groups for all examined indices (Figure 4A and Supplementary Table 4). One-way ANOVA detected significant differences among the sampled groups for Observed ASVs, Chao1, and Shannon diversity (all *p* < 0.001), as well as for the inverse Simpson index (*p* = 0.008). These differences were mainly attributable to the lower diversity of bulk soil compared with the rhizosphere samples. Tukey’s *post hoc* test separated bulk soil from all five accession plots for Observed ASVs, Chao1, and Shannon diversity, whereas differences among the five *M. spicata* accession plots were not significant for these indices.

Fungal alpha diversity did not differ significantly among the sampled groups (Figure 4B, Supplementary Table 5). One-way ANOVA found no significant differences among groups for Observed ASVs (*p* = 0.616), Chao1 richness (*p* = 0.546), Shannon diversity (*p* = 0.593), or inverse Simpson diversity (*p* = 0.700). Mean values varied among accession plots, but within-group variation was high, particularly in B4, J14, and bulk soil.

### 3.6 Bray-Curtis beta diversity

**FIGURE 5:**
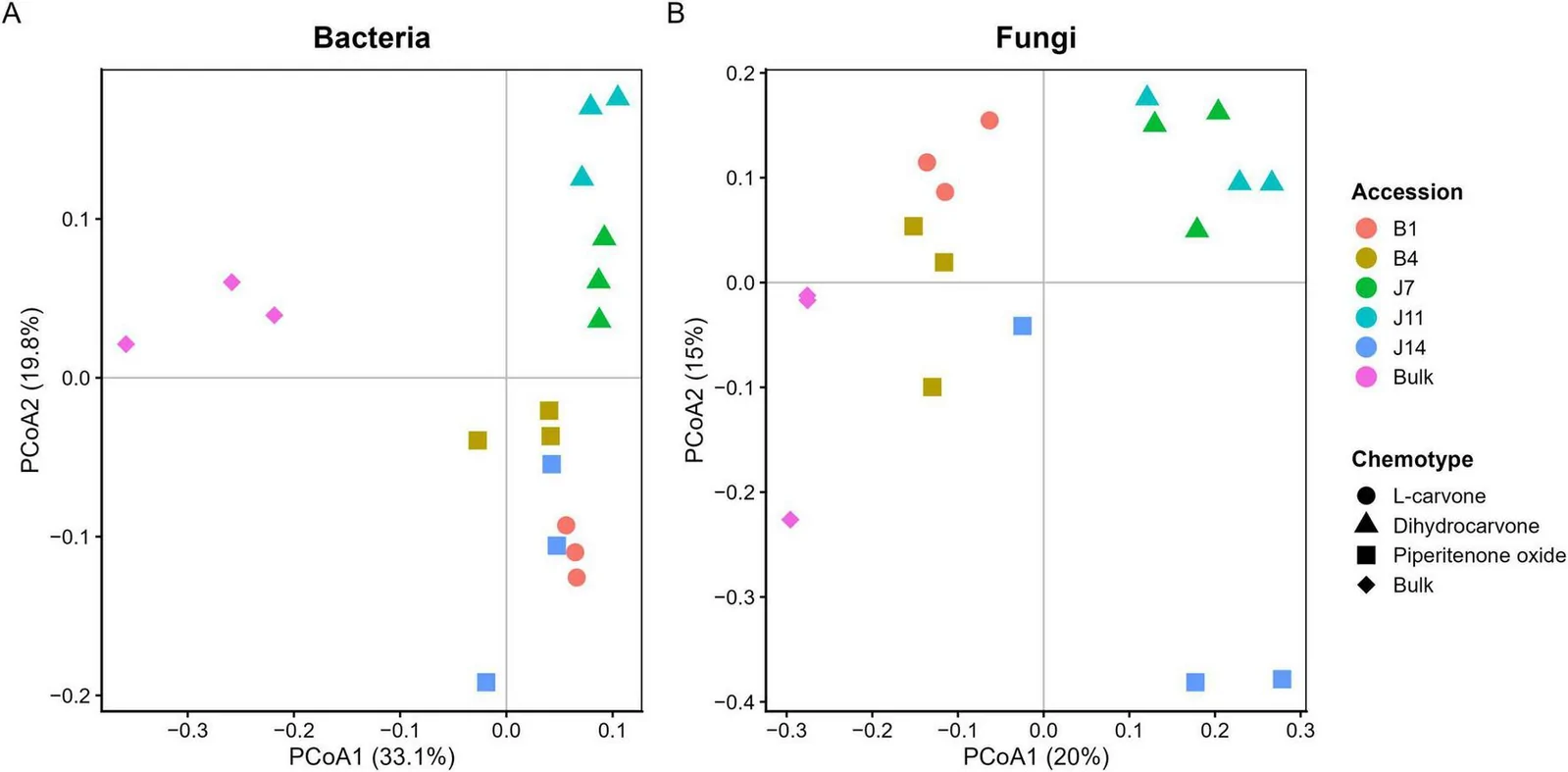
Beta diversity of bacterial **(A)** and fungal **(B)** communities based on Bray-Curtis dissimilarity. Principal coordinate analysis (PCoA) plots show microbial communities based on rarefied ASV abundance data. Colors distinguish the five accessions and bulk soil. Point shapes indicate chemotype for rhizosphere samples, while diamonds represent bulk soil samples. PERMANOVA and PERMDISP were performed with 9999 permutations.

Bray-Curtis-based PCoA showed separation among bacterial sample groups (Figure 5A). The first two PCoA axes explained 33.1 and 19.8% of the total variation, respectively. Bulk soil samples were separated from the rhizosphere samples along PCoA1, indicating a distinct bacterial community composition in bulk soil. Within the rhizosphere samples, structuring associated with the accession plots was also visible. Samples from J7 and J11 (Dihydrocarvone group) tended to separate from the other rhizosphere samples, whereas samples from B1 (L-carvone group) and from B4 and J14 (Piperitenone oxide group) were more closely grouped. PERMANOVA detected significant differences among sample groups in bacterial community composition, explaining 68.71% of the variation in Bray-Curtis dissimilarities (R^2^ = 0.6871, *F* = 5.27, *p* = 0.0001). However, PERMDISP also detected significant differences in multivariate dispersion among bacterial sample groups (*F* = 4.28, *p* = 0.0091), mainly due to the higher dispersion of bulk soil samples.

Fungal beta diversity also differed significantly among the sampled groups, although the ordination pattern was less structured than in the bacterial dataset (Figure 5B). The first two PCoA axes explained 20.0 and 15.0% of the total variation, respectively. Bulk soil samples were positioned toward the negative side of PCoA1, while several rhizosphere samples, particularly those belonging to the Dihydrocarvone group, were located on the positive side. Fungal samples showed greater overall scatter in the ordination than bacterial samples, most notably in J14. PERMANOVA detected significant differences among sample groups in fungal community composition, explaining 54.30% of the total variation (R^2^ = 0.5430, *F* = 2.85, *p* = 0.0001). In contrast to the bacterial dataset, PERMDISP did not detect significant differences in multivariate dispersion among fungal sample groups (*F* = 0.52, *p* = 0.77).

PERMANOVA and PERMDISP were also repeated after excluding bulk soil samples to evaluate differences among the five accession plots. Grouping by accession plot remained significant for both bacterial (R^2^ = 0.6619, *F* = 4.90, *p* = 0.0001) and fungal communities (R^2^ = 0.5060, *F* = 2.56, *p* = 0.0001). No significant differences in multivariate dispersion were detected among the five accession plots in either dataset (bacteria: *F* = 0.30, *p* = 0.8583; fungi: *F* = 0.60, *p* = 0.6811). The subsequent dbRDA analyses were therefore performed on rhizosphere samples only.

### 3.7 Associations of microbial community composition with soil properties and EO profiles

**FIGURE 6:**
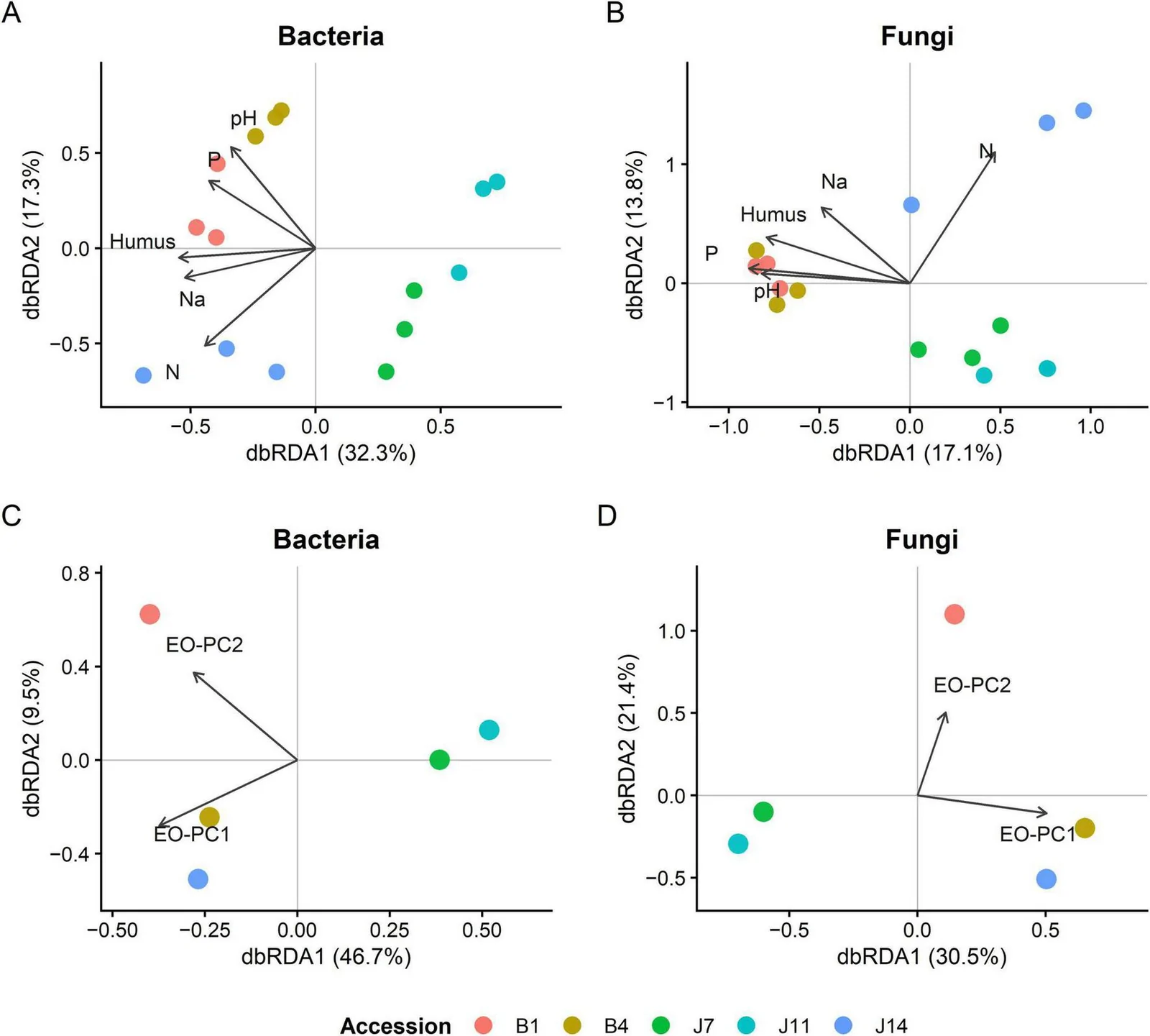
Bray-Curtis-based dbRDA of bacterial and fungal community composition in relation to soil physicochemical variables [**(A)** bacteria; **(B)** fungi] and accession-level aerial-tissue EO profiles [**(C)**, bacteria; **(D)** fungi]. In panels A and B, each point represents an individual rhizosphere sample (*n* = 15) and is colored by accession. Soil vectors indicate the five variables retained in the final models: humus content, pH, nitrate + nitrite nitrogen (N), available sodium (Na), and available phosphorus (P). Available potassium was excluded from the constrained models because of high multicollinearity. In panels **(C,D),** each point represents one accession-level observation (*n* = 5), obtained by averaging microbial relative-abundance profiles across the three within-plot rhizosphere samples and EO composition across the three analytical determinations for each accession. The EO analysis initially included the ten compounds that exceeded 5% relative abundance in at least one EO sample (Figure 1). Hellinger-transformed accession-level EO profiles were reduced by principal component analysis, and EO-PC1 and EO-PC2 were used as explanatory variables in the dbRDA models. Vectors in panels C and D represent EO-PC1 and EO-PC2, the two composite EO-profile gradients used as explanatory variables in the corresponding constrained models. These gradients are derived from EO components measured in aerial plant material and are interpreted as accession-level EO-profile descriptors rather than direct rhizosphere metabolites. Percentages shown on the axes indicate the proportion of total community variation represented by each constrained axis.

Bray-Curtis-based dbRDA was used to evaluate associations between bacterial and fungal community composition and measured soil physicochemical variables in rhizosphere samples from the five *M. spicata* accession plots. The initial variance inflation factor screening identified available potassium as collinear with the other soil variables (VIF = 10.36), and it was therefore excluded from the constrained models. In the final models, VIF values for the five retained variables ranged from 1.14 to 2.38. The bacterial soil model was significant and explained 62.3% of the community variation (adjusted R^2^ = 0.414; *F* = 2.98, *p* = 0.0001; Figure 6A). The first two constrained axes accounted for 32.3 and 17.3% of the total variation, respectively, and both were significant (dbRDA1: *F* = 7.72, *p* = 0.0001; dbRDA2: *F* = 4.58, *p* = 0.0103). Marginal tests identified significant associations with pH (*F* = 3.16, *p* = 0.0053) and nitrate + nitrite nitrogen (*F* = 3.83, *p* = 0.0007), whereas the association with available phosphorus did not reach statistical significance (*F* = 2.11, *p* = 0.0559). Humus content and available sodium were not significant in the marginal tests.

The fungal soil model was also significant, explaining 52.5% of the community variation (adjusted R^2^ = 0.262; *F* = 1.99, *p* = 0.0001; Figure 6B). The first two constrained axes accounted for 17.1% and 13.8% of the total variation and were both significant (dbRDA1: *F* = 3.25, *p* = 0.0003; dbRDA2: *F* = 2.91, *p* = 0.0019). Marginal tests indicated significant associations with humus content (F = 1.62, *p* = 0.0392), pH (*F* = 2.22, *p* = 0.0013), and nitrate + nitrite nitrogen (*F* = 2.13, *p* = 0.0040), while available sodium and phosphorus were not significant.

To examine whether variation in aerial-tissue EO composition was also associated with rhizosphere microbial community structure, a separate accession-level analysis was performed using the ten EO compounds that exceeded 5% relative abundance in at least one sample. PCA summarized variation in these compounds along two dominant compositional gradients. EO-PC1 and EO-PC2 explained 62.5 and 31.5% of the EO-profile variation, respectively, corresponding to 94.0% cumulative variance. EO-PC1 primarily contrasted piperitenone oxide-rich profiles with profiles characterized by cis- and trans-dihydrocarvone, whereas EO-PC2 was associated mainly with L-carvone. The two retained predictors were orthogonal, with VIF values of 1.00 in both microbial models.

The accession-level bacterial dbRDA model was not significant (*F* = 1.28, *p* = 0.342; Figure 6C). The model had R^2^ = 0.562, while adjusted R^2^ was substantially lower (adjusted R^2^ = 0.125). The first two constrained axes accounted for 46.7 and 9.5% of the total bacterial community variation, respectively, and neither axis was significant (dbRDA1: *F* = 2.13, *p* = 0.217; dbRDA2: *F* = 0.65, *p* = 0.667). Marginal tests likewise indicated no significant contribution of EO-PC1 (*F* = 1.52, *p* = 0.325) or EO-PC2 (*F* = 1.05, *p* = 0.433).

The fungal EO model was also not significant (*F* = 1.08, *p* = 0.317; Figure 6D), with R^2^ = 0.520 and adjusted R^2^ = 0.039. The first two constrained axes represented 30.5 and 21.4% of the total fungal community variation, but neither was significant (dbRDA1: *F* = 1.27, *p* = 0.417; dbRDA2: *F* = 1.34, *p* = 0.417). Neither EO-PC1 (*F* = 1.25, *p* = 0.233) nor EO-PC2 (*F* = 0.91, *p* = 0.600) showed a significant marginal association with fungal community composition. These accession-level analyses therefore did not identify a statistically supported association between the dominant gradients of aerial-tissue EO composition and either bacterial or fungal community structure.

## 4 Discussion

This study examined whether *M. spicata* accession plots representing three operational EO-based groups differed in their associated rhizosphere bacterial and fungal communities under similar open-field conditions. Because the accessions were grown in the same experimental field, the system allowed accession-plot-associated microbial patterns to be evaluated in the context of contrasting aerial-tissue EO profiles, while still retaining the natural complexity of field-grown rhizospheres.

### 4.1 EO composition and operational EO-based grouping

Based on their aerial-tissue EO composition, the five accessions were assigned to three operational EO-based groups. J14 and B4 showed piperitenone oxide-dominated profiles (65.2 and 57.5%, respectively), B1 was characterized by an L-carvone-dominated profile (55.0%), and J11 and J7 formed the Dihydrocarvone group, with cis-dihydrocarvone accounting for 39.2 and 32.7% of their EO composition, respectively. Four of the five accessions analyzed in the present study had previously been characterized for EO composition using aerial plant material collected in 2023 (Sfaxi et al., 2025). The dominant EO profiles were highly consistent with those observed here: B4 contained 56.28% piperitenone oxide in the earlier dataset compared with 57.5% in the present study, while J7 contained 32.63% cis-dihydrocarvone and 36.99% trans-dihydrocarvone compared with 32.7 and 35.6%, respectively. B1 was likewise L-carvone-dominated in both datasets, and J14 showed a piperitenone oxide-dominated profile in both sampling years. This agreement between the two sampling years supports the temporal consistency of the dominant EO profiles of these accessions, although slight quantitative variation in individual constituents may occur.

According to the literature data, L-carvone is most frequently given as the main compound of spearmint EO distilled from the leaves, or flowering stems (Baser et al., 1999; Rasooli et al., 2008; Verma et al., 2011; Benomari et al., 2018). Piperitenone oxide (syn. rotundifolone) is a limonene-3-oxo-epoxide derivative and was also described as main EO compound in spearmint (Sartoratto et al., 2004; Koliopoulos et al., 2010); however, its presence is more common in *Mentha suaveolens* (Lawrence, 2007; Benali et al., 2020; Zekri et al., 2023). Dihydrocarvone-type profiles appear to be less common in *M. spicata*, but relatively high proportions of dihydrocarvone-related compounds have been reported previously. According to the summary table of Lawrence (2007) one earlier spearmint dataset contained 21.5% dihydrocarvone and 12.3% dihydrocarvyl acetate, while a Japanese study reported 32.0% cis-dihydrocarvone together with a high proportion of dihydrocarveol (37.8%) in one *M. spicata* sample (Umemoto and Nagasawa, 1980; Kokkini, 1983). Reports specifying trans-dihydrocarvone are more limited; lower amounts have generally been described, or the individual isomer was not specified. A high trans-dihydrocarvone proportion was reported in a PhD thesis, where 21.2% trans-dihydrocarvone occurred together with 21.6% cis-dihydrocarvone (Lawrence, 1978). In the present study, trans-dihydrocarvone reached an even higher level in accession J7 (35.6%), whereas J11 contained a considerable proportion of dihydro-carveol-acetate (24.9%). Although both accessions were assigned to the Dihydrocarvone group, they differed in the relative proportions of dihydrocarvone isomers and related oxygenated derivatives. The presence of dihydrocarvone is more common in horse mint, and the proportion of cis-dihydrocarvone can reach more than 50% (Llorens Molina et al., 2015; Patonay, 2022) in this mint species.

EO compounds detected in aerial tissues should not be interpreted as direct rhizosphere metabolites. Nevertheless, plant volatiles and related terpenoids can also occur belowground; for example, compounds characteristic of pine tissues, including α- and β-pinene and limonene, have been detected in both roots and rhizosphere soil of *Pinus* spp. (Lin et al., 2007). In *M. spicata*, the presence of modified underground stems, or stolons, may provide an additional belowground source of terpenoid compounds, although volatile oil content and composition in spearmint stolons and surrounding soil have not yet been directly characterized. Soil-amendment studies with spearmint residues further support the possibility that *Mentha*-derived secondary metabolites can influence soil processes: incorporation of raw spearmint plant material improved tomato seedling performance and increased soil microbiological activity (Kadoglidou et al., 2014). In a related GC-MS-based study, spearmint-treated soils showed a decline in total essential oil content and in the relative proportion of carvone, whereas some sesquiterpenes, particularly caryophyllene and bourbonene, increased during soil incubation (Karamanoli et al., 2008). These findings do not demonstrate direct transfer of aerial-tissue EO profiles to the rhizosphere, but they support the possibility that spearmint-derived metabolites and residues may contribute to soil microbial community modification.

### 4.2 Microbial diversity and accession-plot-associated community structuring

The sequencing results revealed a clear contrast between the two microbial groups. Bacterial ASV richness was relatively stable across the five accession plots, whereas fungal richness and community composition showed greater among-sample variability.

Alpha diversity showed relatively limited differentiation among the *M. spicata* accession plots. Bacterial richness and diversity differed across the full dataset, but these differences were mainly attributable to the lower diversity of managed inter-row bulk soil. After considering the rhizosphere samples separately, no significant differences among accession plots were detected for Observed ASVs, Chao1 richness or Shannon diversity. Fungal alpha diversity was more variable among individual samples, but none of the examined indices differed significantly among the sampled groups.

Community composition showed clearer spatial structuring than alpha diversity. Bray-Curtis PCoA revealed distinct bacterial patterns among the sampled groups, and the full PERMANOVA explained 68.71% of the variation. Although bacterial dispersion differed when bulk soil was included, this effect was largely associated with the greater dispersion of bulk samples. After bulk soil was excluded, grouping by accession plot remained significant (R^2^ = 0.6619, *p* = 0.0001), while multivariate dispersion no longer differed significantly. Fungal communities were less compact in the ordination, but accession-plot grouping was also significant after exclusion of bulk soil (R^2^ = 0.5060, *p* = 0.0001), with no evidence of unequal dispersion.

These results indicate that differences among the sampled accession plots were expressed mainly in community composition rather than in overall richness or diversity. Such compositional shifts without corresponding changes in alpha diversity are consistent with the broader view that plant-associated factors and local soil conditions can alter rhizosphere community assembly without necessarily changing total microbial diversity (Bulgarelli et al., 2013; Philippot et al., 2013; Trivedi et al., 2020). Comparable plant-associated rhizosphere differentiation has been reported for other *Lamiaceae* and medicinal plants, including oregano, peppermint, thyme, sage and lavender (Applebaum et al., 2022; Zharkova et al., 2023; Deng et al., 2024; Steinberger et al., 2024; Li L. et al., 2025). Bacterial communities formed more compact accession-plot patterns than fungi, whereas fungal richness, taxonomic composition and ordination showed greater within-plot variability. The latter is compatible with the spatially heterogeneous ecology of soil fungi, including variation associated with hyphal growth, spore distribution, microsite structure and contrasting ecological guilds (Tedersoo et al., 2014; Applebaum et al., 2022; Deng et al., 2024).

### 4.3 Taxonomic composition across *M. spicata* accession plots

The dominant bacterial and fungal groups were typical of soil and rhizosphere communities. Actinomycetota, Pseudomonadota, Chloroflexota, Acidobacteriota and Bacillota together represented most of the bacterial community, while Ascomycota dominated the fungal dataset, followed by Mortierellomycota and Basidiomycota. Similar high-level taxonomic patterns are widely reported in soil and rhizosphere microbiomes and reflect the combined influence of edaphic conditions and plant-associated processes (Fierer and Jackson, 2006; Philippot et al., 2013; Tedersoo et al., 2014; Fierer, 2017). Comparable dominant groups have also been reported in rhizosphere studies of *Lamiaceae* and other medicinal plants (Zharkova et al., 2023; Deng et al., 2024; Li L. et al., 2025).

Several descriptive differences accompanied the community-level separation. Bulk soil contained higher proportions of Actinomycetota and Chloroflexota and lower proportions of Acidobacteriota and Bacillota than the rhizosphere samples. Among the accession plots, J7 and J11 showed the highest Bacillota abundances, and J7 also had the highest proportion of *Bacillaceae*. Fungal composition varied more strongly among samples: J14 contained the highest proportion of Ascomycota, B1 showed relatively high Mortierellomycota abundance, and Basidiomycota was most abundant in J7 and J11. Family-level profiles likewise differed among accession plots, particularly for Nectriaceae, Stachybotryaceae, Didymellaceae and other abundant fungal families. These taxonomic patterns provide descriptive support for the beta-diversity differences but should not be interpreted as accession-specific biomarkers because the accession treatments were not independently replicated at the plot level.

### 4.4 Soil and aerial-tissue EO profiles in relation to microbial community structure

Local soil chemistry was significantly associated with both bacterial and fungal community composition. After removal of available potassium because of high multicollinearity, the bacterial dbRDA model explained 62.3% of community variation (adjusted R^2^ = 0.414, *p* = 0.0001). Marginal tests identified significant associations with pH and nitrate + nitrite nitrogen, while available phosphorus approached but did not reach statistical significance. The fungal model explained 52.5% of the variation (adjusted R^2^ = 0.262, *p* = 0.0001), with significant marginal associations for humus content, pH and nitrate + nitrite nitrogen. The importance of pH is notable because its measured range was narrow, suggesting that even relatively small differences in soil conditions were associated with detectable community shifts. Edaphic control of bacterial and fungal community structure, particularly through pH, organic matter and nutrient availability, is well established in soil microbiome studies (Fierer and Jackson, 2006; Rousk et al., 2010; Bulgarelli et al., 2013; Fierer, 2017). In the present field system, these relationships indicate that local soil heterogeneity contributed substantially to the microbial differences observed among accession plots.

The relationship with aerial-tissue EO composition was less clear. PCA summarized variation in the ten major EO components along two orthogonal gradients that together represented 94.0% of EO-profile variation. When microbial and EO data were analyzed at the corresponding accession level, neither the bacterial nor the fungal dbRDA model was significant. The bacterial model retained a relatively high unadjusted R^2^ (0.562), but adjusted R^2^ decreased to 0.125 (*p* = 0.342); the fungal model showed a similar contrast between unadjusted R^2^ (0.520) and adjusted R^2^ (0.039; *p* = 0.317). The present data therefore do not provide statistical support for an association between the dominant aerial-tissue EO gradients and rhizosphere community composition.

Secondary metabolites can influence plant-associated microbial communities, and aromatic plant chemistry has been linked to microbial selection in several systems (Checcucci et al., 2017; Castronovo et al., 2020; Trivedi et al., 2020; Pang et al., 2021; Koprivova and Kopriva, 2022; Semenzato et al., 2023). Experiments with *M. spicata* and *M.* × *piperita* residues have also shown that mint-derived material can alter soil microbial activity and community composition and that EO composition changes rapidly during decomposition (Karamanoli et al., 2018; Ainalidou et al., 2021). These studies support the biological plausibility of links between aromatic plant chemistry and soil microorganisms, but they do not establish such a mechanism in the present field experiment. Here, EO profiles were measured from pooled aerial tissues rather than from roots, exudates or rhizosphere soil, and the nonsignificant accession-level EO models do not support attribution of the microbial differences to EO composition.

### 4.5 Limitations and implications

The main limitation of this study is the lack of independent plot-level replication, as each accession was represented by a single field plot and the three soil samples reflected within-plot spatial variation rather than independent biological replicates. The study was also restricted to one field site and one phenological stage. Because sampling was performed at a single time point, the observed bacterial and fungal community compositions represent a temporal snapshot, and potential seasonal and plant age- or developmental-stage-related changes could not be evaluated. In addition, accession identity and operational EO-based grouping cannot be separated, and the EO analysis was based on five accession-level aerial-tissue profiles rather than direct measurements of root exudates or rhizosphere metabolites. Future studies should therefore include independently replicated accession plots, repeated sampling, and direct characterization of belowground plant metabolites to better resolve plant-, soil-, and EO-related contributions to rhizosphere microbiome structure.

## 5 Conclusion

This study showed that field-grown *Mentha spicata* accession plots with contrasting aerial-tissue EO profiles harbored differentiated rhizosphere bacterial and fungal communities. Alpha-diversity differences were limited, whereas Bray-Curtis analyses revealed significant accession-plot-associated structuring in both microbial groups. Soil physicochemical properties, particularly pH and nitrate + nitrite nitrogen, were significantly associated with community composition, while accession-level aerial-tissue EO profiles showed no statistically supported relationship with either bacterial or fungal communities. These findings emphasize the importance of local soil conditions and accession-plot-associated variation in *M. spicata* rhizosphere ecology and provide a basis for replicated studies incorporating direct measurements of belowground plant metabolites.

## Data Availability

The datasets presented in this study can be found in online repositories. The names of the repository/repositories and accession number(s) can be found in the article/Supplementary material.
