# Supplementary material for: Rhizosphere bacterial and fungal community structure varies among field-grown Mentha spicata accession plots with different essential oil profiles
Source: Front Microbiol. 2026 Sep 3;17:1939529. doi: 10.3389/fmicb.2026.1939529 (PMC13582204; doi:10.3389/fmicb.2026.1939529)
Supplement: Supplementary file 1 [file Table_1.DOCX]

Supplementary Material

The operational EO-based groups used throughout the Supplementary Material are B1 (L-carvone group), B4 and J14 (Piperitenone oxide group), and J7 and J11 (Dihydrocarvone group).

**Supplementary Table S1** Essential oil composition of the analyzed *M. spicata* accessions

| **Compound** | **RT^a^** | **LRI^b^** | **A. LRI^c^** | **B4** | **B1** | **J11** | **J7** | **J14** |
| --- | --- | --- | --- | --- | --- | --- | --- | --- |
| α-Pinene | 5.56 | 938 | 932 | 0.44±0.06 | 0.39±0.18 | 0.16±0.08 | 0.20±0.13 | 0.29±0.05 |
| Sabinene | 6.52 | 976 | 969 | 0.91±0.01 | 0.78±0.13 | 0.18±0.08 | 0.34±0.12 | 0.57±0.08 |
| β-Pinene | 6.64 | 981 | 974 | 1.68±0.11 | 1.03±0.31 | 0.34±0.14 | 0.71±0.26 | 1.16±0.12 |
| 3-Octanol | 6.88 | 990 | 982 | 0.34±0.06 | 0.00±0.00 | 0.00±0.00 | 0.00±0.00 | 0.19±0.02 |
| β-Myrcene | 6.99 | 995 | 988 | 7.40±0.57 | 1.74±0.20 | 2.02±0.29 | 0.50±0.08 | 5.27±0.31 |
| Limonene | 8.19 | 1029 | 1024 | 6.67±0.93 | 8.57±1.83 | 4.69±0.38 | 5.64±0.55 | 6.02±0.40 |
| 1,8-Cineole | 8.38 | 1034 | 1026 | 14.88±0.74 | 8.58±0.87 | 2.46±0.33 | 1.23±0.11 | 11.99±0.67 |
| (E)-Ocymene (trans-beta-ocymene) | 8.85 | 1046 | 1044 | 0.28±0.01 | 0.19±0.01 | 0.00±0.00 | 0.24±0.01 | 0.49±0.08 |
| γ-Terpinene | 9.20 | 1056 | 1054 | 0.56±0.23 | 0.42±0.08 | 0.00±0.00 | 0.00±0.00 | 0.49±0.11 |
| Trans-sabinene-hydrate | 9.73 | 1070 | 1098 | 0.00±0.00 | 10.87±0.21 | 0.00±0.00 | 0.00±0.00 | 0.00±0.00 |
| α-Terpinolene | 10.29 | 1085 | 1086 | 0.00±0.00 | 0.15±0.06 | 0.00±0.00 | 0.00±0.00 | 0.00±0.00 |
| Linalool | 10.76 | 1097 | 1095 | 0.12±0.02 | 0.00±0.00 | 0.00±0.00 | 0.00±0.00 | 0.10±0.02 |
| Isopentyl isovalerate | 10.94 | 1102 | 1102 | 0.12±0.02 | 0.00±0.00 | 0.00±0.00 | 0.00±0.00 | 0.00±0.00 |
| Octen-3-yl acetate | 11.19 | 1108 | 1110 | 0.15±0.01 | 0.00±0.00 | 0.00±0.00 | 0.00±0.00 | 0.00±0.00 |
| α-Terpineol (cis-dehydro) | 13.49 | 1164 | 1143 | 0.75±0.08 | 0.44±0.19 | 0.00±0.00 | 0.00±0.00 | 0.39±0.07 |
| Terpinene-4-ol | 13.96 | 1175 | 1174 | 0.20±0.12 | 1.38±0.45 | 0.00±0.00 | 0.00±0.00 | 0.00±0.00 |
| α-Terpineol | 14.55 | 1189 | 1186 | 0.15±0.03 | 0.62±0.14 | 0.00±0.00 | 0.00±0.00 | 0.00±0.00 |
| Dihydro-carveol | 14.68 | 1192 | 1192 | 0.00±0.00 | 0.67±0.26 | 0.00±0.00 | 0.00±0.00 | 0.00±0.00 |
| Cis-dihydrocarvone | 14.74 | 1194 | 1191 | 0.00±0.00 | 0.79±0.12 | 39.21±1.23 | 32.74±1.00 | 0.00±0.00 |
| Myrtenol | 14.78 | 1194 | 1194 | 0.22±0.10 | 0.00±0.00 | 0.00±0.00 | 0.00±0.00 | 0.00±0.00 |
| Trans-dihydrocarvone | 15.12 | 1203 | 1200 | 0.00±0.00 | 0.00±0.00 | 8.58±0.29 | 35.61±0.26 | 0.00±0.00 |
| Hexenyl 2-methyl butanoat (3Z) | 16.20 | 1228 | 1229 | 0.13±0.04 | 0.00±0.00 | 0.00±0.00 | 0.00±0.00 | 0.00±0.00 |
| Cis-carveol | 16.31 | 1231 | 1215 | 0.00±0.00 | 0.00±0.00 | 0.49±0.08 | 1.98±0.37 | 0.00±0.00 |
| Pulegon | 16.50 | 1236 | 1233 | 0.00±0.00 | 0.00±0.00 | 0.12±0.00 | 0.00±0.00 | 0.00±0.00 |
| L-carvone | 16.71 | 1241 | 1239 | 0.00±0.00 | 54.98±2.03 | 0.71±0.06 | 0.33±0.03 | 0.00±0.00 |
| Piperitone | 17.08 | 1249 | 1249 | 0.00±0.00 | 0.00±0.00 | 0.00±0.00 | 0.10±0.00 | 0.00±0.00 |
| Dihydro-carveol-acetate (iso) | 20.11 | 1324 | 1312 | 0.00±0.00 | 0.31±0.18 | 24.95±3.52 | 1.54±0.04 | 0.00±0.00 |
| Piperitenone | 20.94 | 1348 | 1340 | 0.00±0.00 | 0.00±0.00 | 0.00±0.00 | 3.21±0.07 | 0.00±0.00 |
| Dihydro-carveol-acetate (neoiso) | 21.57 | 1364 | 1356 | 0.00±0.00 | 0.00±0.00 | 6.63±1.06 | 1.57±0.17 | 0.00±0.00 |
| Piperitenone oxide | 21.97 | 1375 | 1366 | 57.52±2.13 | 0.00±0.00 | 0.00±0.00 | 0.00±0.00 | 65.15±2.66 |
| β-Bourbonene | 22.26 | 1383 | 1387 | 0.10±0.00 | 1.32±0.30 | 0.28±0.08 | 0.00±0.00 | 0.00±0.00 |
| β-Elemene | 22.55 | 1391 | 1389 | 0.00±0.00 | 0.39±0.17 | 1.61±0.29 | 0.13±0.01 | 0.00±0.00 |
| Cis-jasmone | 23.04 | 1404 | 1392 | 0.46±0.03 | 0.00±0.00 | 0.00±0.00 | 0.00±0.00 | 0.00±0.00 |
| β-Caryophyllene | 23.68 | 1420 | 1417 | 2.52±0.07 | 1.75±0.37 | 3.61±0.56 | 4.85±0.18 | 4.85±0.22 |
| α-Humulene | 25.07 | 1454 | 1452 | 0.00±0.00 | 0.00±0.00 | 0.00±0.00 | 0.13±0.00 | 0.00±0.00 |
| β-farnesene | 25.27 | 1459 | 1454 | 0.17±0.01 | 0.00±0.00 | 0.00±0.00 | 0.00±0.00 | 0.00±0.00 |
| Germacrene-D | 26.18 | 1482 | 1480 | 3.37±0.60 | 0.83±0.37 | 2.50±0.48 | 4.00±0.11 | 1.04±0.09 |
| Bicyclogermacrene | 26.81 | 1497 | 1500 | 0.00±0.00 | 0.29±0.18 | 0.00±0.00 | 0.79±0.01 | 0.00±0.00 |
| δ-Cadinene | 27.08 | 1524 | 1513 | 0.00±0.00 | 0.17±0.09 | 0.00±0.00 | 0.45±0.01 | 0.56±0.10 |
| Caryophyllene-oxide | 30.20 | 1590 | 1582 | 0.00±0.00 | 0.00±0.00 | 0.00±0.00 | 0.00±0.00 | 0.38±0.02 |
| α-Cadinol | 32.77 | 1658 | 1652 | 0.00±0.00 | 0.00±0.00 | 0.00±0.00 | 0.11±0.00 | 0.00±0.00 |
| Sum: |  |  |  | 99.08 | 97.50 | 98.56 | 97.44 | 98.94 |

Values represent mean relative percentages ± SD from three hydrodistillations of pooled material from each accession plot (n = 3).

^a^RT: Retention Time; ^b^LRI: Linear Retention Index; ^c^A. LRI: Adams Linear Retention Index

**Supplementary Table S2.** Summary of bacterial 16S rRNA gene sequencing data and taxonomic richness across *M. spicata* rhizosphere and bulk soil samples

| **Samples** | **Nb domain** | **Nb phylum** | **Nb class** | **Nb order** | **Nb family** | **Nb genus** | **Nb species** | **Nb asv** | **Nb sequences** |
| --- | --- | --- | --- | --- | --- | --- | --- | --- | --- |
| B1a | 1 | 28 | 65 | 135 | 200 | 419 | 513 | 3080 | 121161 |
| B1b | 1 | 27 | 64 | 134 | 199 | 423 | 515 | 3079 | 136310 |
| B1c | 1 | 28 | 65 | 136 | 202 | 424 | 518 | 3100 | 129611 |
| B4a | 1 | 27 | 64 | 132 | 196 | 410 | 500 | 3049 | 160205 |
| B4b | 1 | 27 | 64 | 135 | 200 | 421 | 512 | 3088 | 125984 |
| B4c | 1 | 28 | 65 | 136 | 202 | 422 | 514 | 3077 | 107795 |
| J7a | 1 | 28 | 65 | 136 | 204 | 426 | 519 | 3122 | 164787 |
| J7b | 1 | 28 | 65 | 135 | 203 | 426 | 516 | 3105 | 116706 |
| J7c | 1 | 28 | 65 | 136 | 203 | 427 | 519 | 3101 | 153340 |
| J11a | 1 | 28 | 65 | 136 | 203 | 423 | 510 | 3089 | 168290 |
| J11b | 1 | 28 | 65 | 136 | 202 | 423 | 512 | 3073 | 156415 |
| J11c | 1 | 28 | 65 | 136 | 203 | 423 | 512 | 3079 | 143208 |
| J14a | 1 | 28 | 65 | 135 | 201 | 425 | 517 | 3099 | 123541 |
| J14b | 1 | 27 | 64 | 130 | 193 | 398 | 483 | 2973 | 137643 |
| J14c | 1 | 28 | 65 | 136 | 201 | 426 | 519 | 3111 | 147977 |
| Bulk_a | 1 | 24 | 60 | 123 | 182 | 376 | 458 | 2648 | 147840 |
| Bulk_b | 1 | 26 | 63 | 129 | 192 | 397 | 482 | 2872 | 138224 |
| Bulk_c | 1 | 27 | 64 | 131 | 194 | 395 | 473 | 2817 | 168574 |
| **Total** | **1** | **28** | **65** | **136** | **204** | **435** | **527** | **3157** | **2547611** |

“Total” indicates the combined values across all samples. For sequence reads, values represent the sum across samples; for taxonomic groups and ASVs, values represent the total number detected in the full dataset. The suffixes a–c denote the three within-plot subsamples collected from each accession plot, whereas Bulk_a–Bulk_c denote the three individual bulk soil samples.

**Supplementary Table S3.** Summary of fungal ITS gene sequencing data and taxonomic richness across *M. spicata* rhizosphere and bulk soil samples

| **Samples** | **Nb domain** | **Nb phylum** | **Nb class** | **Nb order** | **Nb family** | **Nb genus** | **Nb species** | **Nb asv** | **Nb sequences** |
| --- | --- | --- | --- | --- | --- | --- | --- | --- | --- |
| B1a | 1 | 9 | 30 | 52 | 99 | 168 | 219 | 425 | 90335 |
| B1b | 1 | 10 | 30 | 53 | 98 | 166 | 227 | 451 | 75393 |
| B1c | 1 | 10 | 33 | 55 | 96 | 152 | 204 | 407 | 109657 |
| B4a | 1 | 9 | 25 | 44 | 79 | 121 | 138 | 240 | 114756 |
| B4b | 1 | 10 | 34 | 57 | 99 | 164 | 211 | 429 | 127103 |
| B4c | 1 | 11 | 35 | 57 | 99 | 164 | 219 | 445 | 86501 |
| J7a | 1 | 10 | 34 | 56 | 103 | 162 | 213 | 434 | 96949 |
| J7b | 1 | 10 | 30 | 49 | 86 | 145 | 190 | 396 | 64128 |
| J7c | 1 | 11 | 33 | 56 | 96 | 160 | 216 | 429 | 96772 |
| J11a | 1 | 9 | 26 | 43 | 81 | 136 | 179 | 345 | 115918 |
| J11b | 1 | 11 | 29 | 49 | 83 | 136 | 185 | 367 | 96075 |
| J11c | 1 | 9 | 27 | 46 | 79 | 133 | 176 | 321 | 117573 |
| J14a | 1 | 5 | 18 | 36 | 73 | 129 | 173 | 321 | 119805 |
| J14b | 1 | 5 | 15 | 35 | 66 | 121 | 153 | 284 | 58383 |
| J14c | 1 | 10 | 36 | 56 | 106 | 185 | 246 | 478 | 82458 |
| Bulk_a | 1 | 10 | 29 | 50 | 90 | 154 | 203 | 411 | 93858 |
| Bulk_b | 1 | 7 | 19 | 32 | 61 | 99 | 127 | 247 | 126346 |
| Bulk_c | 1 | 11 | 33 | 54 | 94 | 156 | 204 | 423 | 124814 |
| **Total** | **1** | **12** | **43** | **81** | **156** | **272** | **379** | **837** | **1796824** |

“Total” indicates the combined values across all samples. For sequence reads, values represent the sum across samples; for taxonomic groups and ASVs, values represent the total number detected in the full dataset. The suffixes a–c denote the three within-plot subsamples collected from each accession plot, whereas Bulk_a–Bulk_c denote the three individual bulk soil samples.

**Supplementary Table S4.** Alpha diversity indices of bacterial communities across the five *M. spicata* accession plots and bulk soil

| **Sample group** | **Observed** | **Chao1** | **Shannon** | **InvSimpson** |
| --- | --- | --- | --- | --- |
| B1 | 3071.33±15.50a | 3103.99±15.29a | 7.275±0.019a | 692.20±29.38a |
| B4 | 3059.67±31.77a | 3088.57±25.64a | 7.226±0.035a | 615.29±61.20a |
| J7 | 3095.67±14.47a | 3113.53±11.58a | 7.257±0.056a | 541.55±162.24ab |
| J11 | 3055.00±6.24a | 3093.30±9.07a | 7.228±0.032a | 627.77±46.89a |
| J14 | 3040.67±93.40a | 3081.27±60.67a | 7.225±0.099a | 646.02±67.65a |
| Bulk | 2740.33±115.29b | 2821.01±118.42b | 6.889±0.120b | 382.18±50.78b |
| Global p | *p* < 0.001 | *p* < 0.001 | *p* < 0.001 | *p* = 0.008 |

Values represent means ± standard deviation based on three soil samples for each accession plot and for bulk soil (n = 3). Different lowercase letters within a column indicate significant differences among groups according to Tukey’s HSD test following one-way ANOVA. Global *p* values correspond to the one-way ANOVA.

**Supplementary Table S5.** Alpha diversity indices of fungal communities across the five *M. spicata* accession plots and bulk soil

| **Sample group** | **Observed** | **Chao1** | **Shannon** | **InvSimpson** |
| --- | --- | --- | --- | --- |
| B1 | 422.00±21.07 | 435.03±28.38 | 4.525±0.142 | 43.58±9.02 |
| B4 | 353.00±113.45 | 368.85±110.95 | 4.405±0.457 | 42.62±22.66 |
| J7 | 412.33±17.21 | 438.77±25.59 | 4.339±0.093 | 36.91±7.72 |
| J11 | 331.33±22.55 | 355.52±25.50 | 4.210±0.163 | 31.87±6.96 |
| J14 | 354.33±100.15 | 383.85±79.96 | 4.322±0.378 | 41.47±20.46 |
| Bulk | 344.67±106.27 | 362.43±96.46 | 4.159±0.062 | 28.64±3.53 |
| Global p | *p* = 0.616 | *p* = 0.546 | *p* = 0.593 | *p* = 0.700 |

Values represent means ± standard deviation based on three soil samples for each accession plot and for bulk soil (n = 3). There were no significant differences among groups according to Tukey’s HSD test following one-way ANOVA. Global *p* values correspond to the one-way ANOVA.
